# Safety evaluation of the atmospheric low-temperature plasma (ALTP) on the conjunctiva: an animal study and histopathological findings; 6-month follow-up

**DOI:** 10.1186/s12886-021-02053-8

**Published:** 2021-09-13

**Authors:** Farhad Nejat, Khosrow Jadidi, Fahimeh Asadi Amoli, Shiva Bagheri, Hossein Aghamollaei, Mohammad-Amin Nejat, Nazanin-Sadat Nabavi, Shima Eghtedari

**Affiliations:** 1 Vision Health Research Center, Semnan University of Medical Science, Semnan, Iran; 2 Department of Pathology, Tehran University of Medical Sciences, Tehran, Iran

**Keywords:** Atmospheric-pressure low-temperature plasma, Conjunctival tissue, Soft surgery

## Abstract

**Background:**

Plasma medicine is an innovative research field focused on the application of atmospheric-pressure low-temperature plasmas (ALTP) for therapeutic purposes. Considering the potentials of plasma in ophthalmology, in this study, we evaluated the safety of plasma on the conjunctival tissue in animal models for 6 months.

**Methods:**

Twelve adult male New Zealand albino rabbits were divided into four groups. The right eye of each rabbit was chosen for the test and the left eye was considered as the control. Experiments were performed using the Plexr device (GMV, Rocca Priora, RM, Italy). Four plasma spots were applied on the superior part of the conjunctiva (from 10 to 2 o’clock positions) using the continuous mode and a low power level (white handpiece) of the Plexr. For evaluation of the plasma safety, the histopathological changes were assessed 1 week (A), 1 month (B), 3 months (C), and 6 months (D) after the intervention.

**Results:**

According to the histopathological findings, a mild decrease in blood vessels and severe stromal edema, as well as a superficial epithelium loss, were observed in group A. No chronic inflammation, scar tissue, deposition, and hemorrhage were found in group B. Epithelialization was confirmed by the histological examinations after 1 month. There was no evidence of atypia or dysplasia after 3 and 6 months.

**Conclusion:**

In conclusion, there were no persistent histopathological changes on conjunctival tissue after plasma exposure. Then, plasma can be considered as a minimally invasive alternative method for treating some ocular surface disorders.

**Supplementary Information:**

The online version contains supplementary material available at 10.1186/s12886-021-02053-8.

## Background

In recent decades, plasma medicine has been considered a multidisciplinary field of research combining plasma physics, engineering, life science, and clinical medicine [1]. Plasma is often defined as the fourth state of matter following solid, liquid, and gaseous states. It can be generated by supplying energy to a neutral gas until an ionized gaseous composed of ions, electrons, and photons is achieved [2, 3]. Plasmas are classified as either thermal plasma or non-thermal plasma [4]. Application of plasma in the medicine started in the early- and mid-1990s by the generation of atmospheric-pressure low-temperature plasmas (ALTP) [5]. At present, plasma medicine is mainly focused on treating living cells, tissues, and surface modifications [1, 6]. To date, there are several types of plasma generators applied for medical practices. A growingly one is Plexr - a cordless micro-surgical handheld device. The usage of the Plexr (GMV, Rocca Priora, RM, Italy) as a minimally invasive soft surgery device has been increasing over recent years. Its application has emerged in various medical disciplines of aesthetic medicine, oculoplastic surgery, and dentistry [7–11]. This apparatus ionizes the air between the handpiece tip and the target tissue by generating an appropriate potential difference. The created energy is transferred onto the superficial layer of the tissue. Then, it sublimates the area where the plasma is applied. The energy is not delivered directly from the device to the deeper or surrounding tissues [12].

Considering the potential of the Plexr in medical applications, determination of the safety profile of ALTP for various applications, including the ocular structure, seemed necessary. Accordingly, Nejat et.al (2019) assessed the effects of Plexr on the ocular surface in animal models [13]. They evaluated the histopathological changes on the conjunctiva after 1 month of plasma exposure. Their results showed no obvious pathological subsequences, including inflammation, scar tissue, deposition, and hemorrhage after 1 month.

In continuation of the previous studies, the present study aimed to investigate the histopathological influences of plasma exposure on the conjunctival surface after 6 months of intervention. This knowledge can help to treat a wide range of ocular surface disorders including conjunctival cyst, pinguecula, and conjunctivochalasis (CCh) with minimally invasive plasma soft surgery.

## Methods

This experimental study involved twelve adult male New Zealand albino rabbits each weighing 2–2.5 kg and was performed in the Vision Health Ophthalmic Center, Tehran, Iran. This animal study was performed with adherence to the tenets of the Declaration of Helsinki and the ‘Statement for the Use of Animals in Ophthalmic and Visual Research’ from the Association for Research in Vision and Ophthalmology (ARVO). This study was also approved by the Institutional Animal Care and Use Committee of the University of Medical Sciences of Semnan, Iran.

Rabbits were divided into four groups (A to D) each containing 3 rabbits. Rabbits were housed in separate cages under standard conditions including the temperature of 25–28 °C, 50–60% humidity, and 12/12 h light-dark cycles. Before initiating the study, rabbits’ eyes were evaluated clinically for eye complications. An intraperitoneal injection of 44 mg/Kg ketamine hydrochloride (Ketaset, 100 mg/ml, Pfizer, NY, USA) and 6–8 mg/Kg xylazine (Chanazine, 20 mg/ml, Chanelle Pharma, IL, USA) solution in the sterile normal saline was used to anesthetize rabbits.

**Fig. 1:**
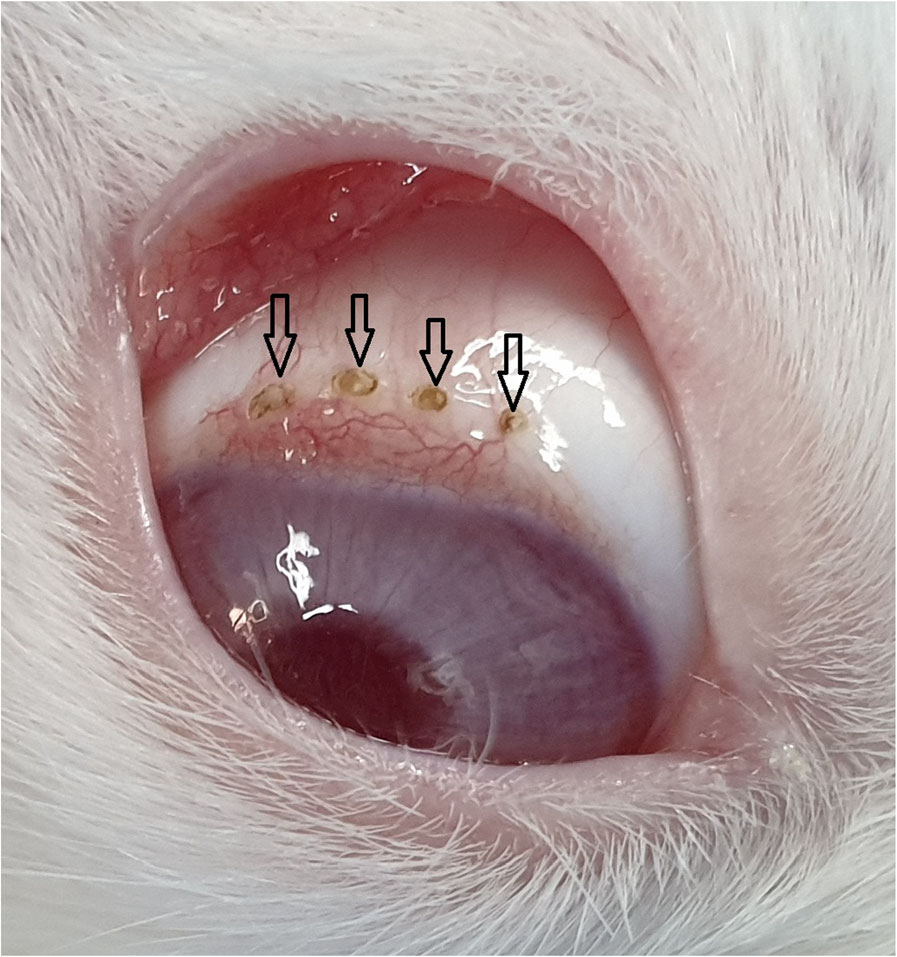
The atmospheric-pressure low-temperature plasma spots were applied on four sites in an arc a few millimeters behind the limbus on the superior bulbar conjunctiva (from 10 to 2 o’clock positions on the right eye)

Experiments were performed using the Plexr device with continuous mode and in low power level (white handpiece, Vpp = 500 V, power = 0.7 W, and frequency = 75 kHz) (Table 1). It was used at 0.7-s intervals using a 22-gauge needle. Plasma spots were applied on four areas located at the superior part of the conjunctiva (from 10 to 2 o’clock position on the right eye) (Fig. 1). The left eye was left with no intervention as the control in all rabbits. Topical chloramphenicol 0.5% (Sina Darou, Tehran, Iran) was used every 6 h for 1 week to prevent infection.

**Table 1:**
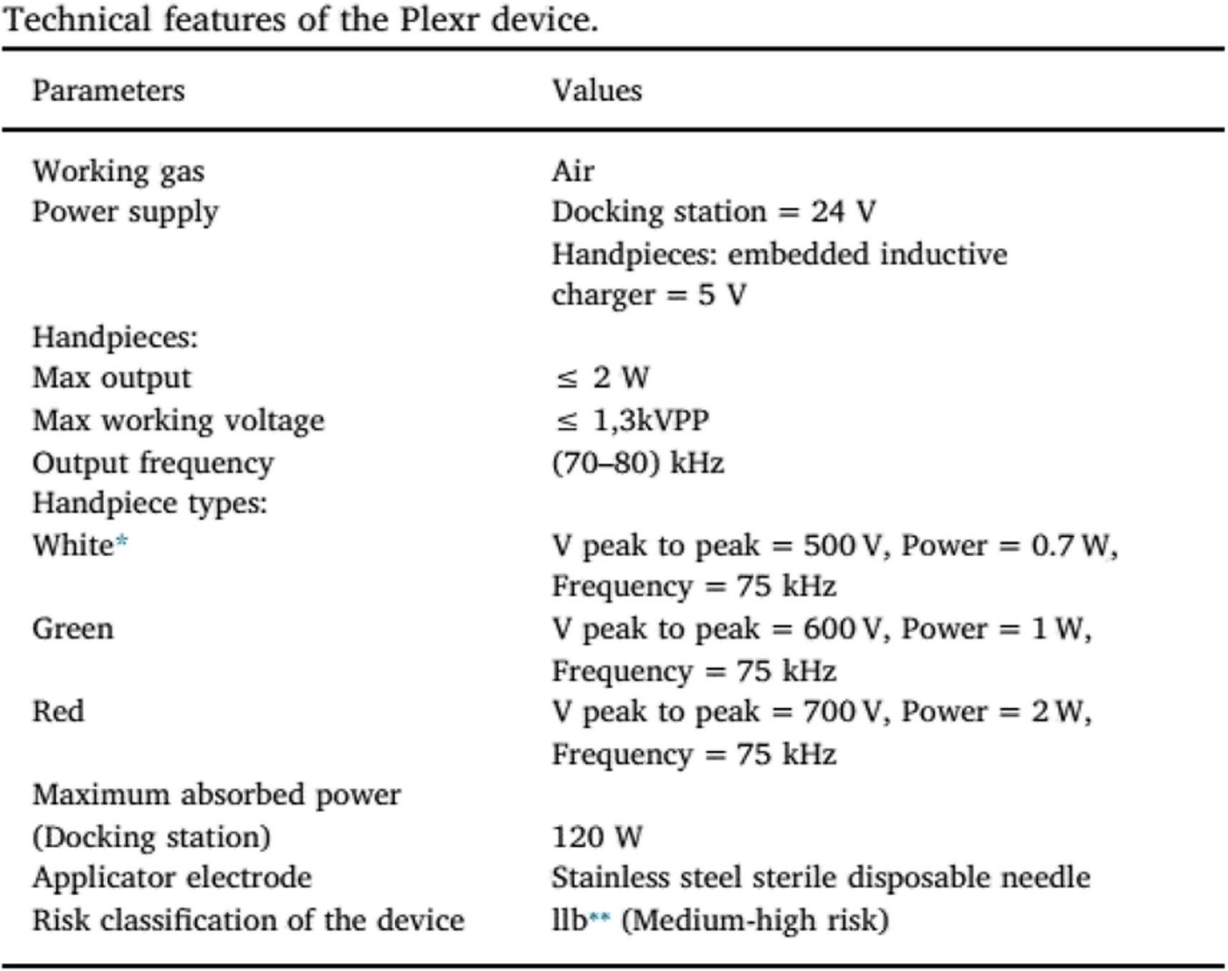
Technical features of the Plexr device

^*^In the current study, the white handpiece was used.

^**^This classification relates to the non-invasive medical devices within the field of dermatology.

### Examination

#### External examination

After 1 week (group A), 1 month (group B), 3 months (group C) and, 6 months (group D) exposure to plasma, the Rabbits’ eyes were evaluated clinically for corneal haze, conjunctival chemosis, redness, discharge, and lid swelling.

#### Histopathological examination

To evaluate the effects of plasma on the exposed surface of the conjunctiva, the histopathological examinations characterized by epithelial loss, hemorrhage, inflammation, scarring, and swelling were performed in the above-mentioned groups.

For this purpose, animals were first sacrificed under deep general anesthesia (using double the dose of intravenous ketamine and xylazine). The eyes of rabbits were enucleated 1 week, 1 month, 3 months, and 6 months after the intervention. Then, specimens were immediately fixed in 10% formalin and processed to obtain 4-μm microscopic sections for histological analysis. Sections were stained by hematoxylin and eosin (H&E method) and were evaluated under a light microscope.

## Results

**Fig. 2:**
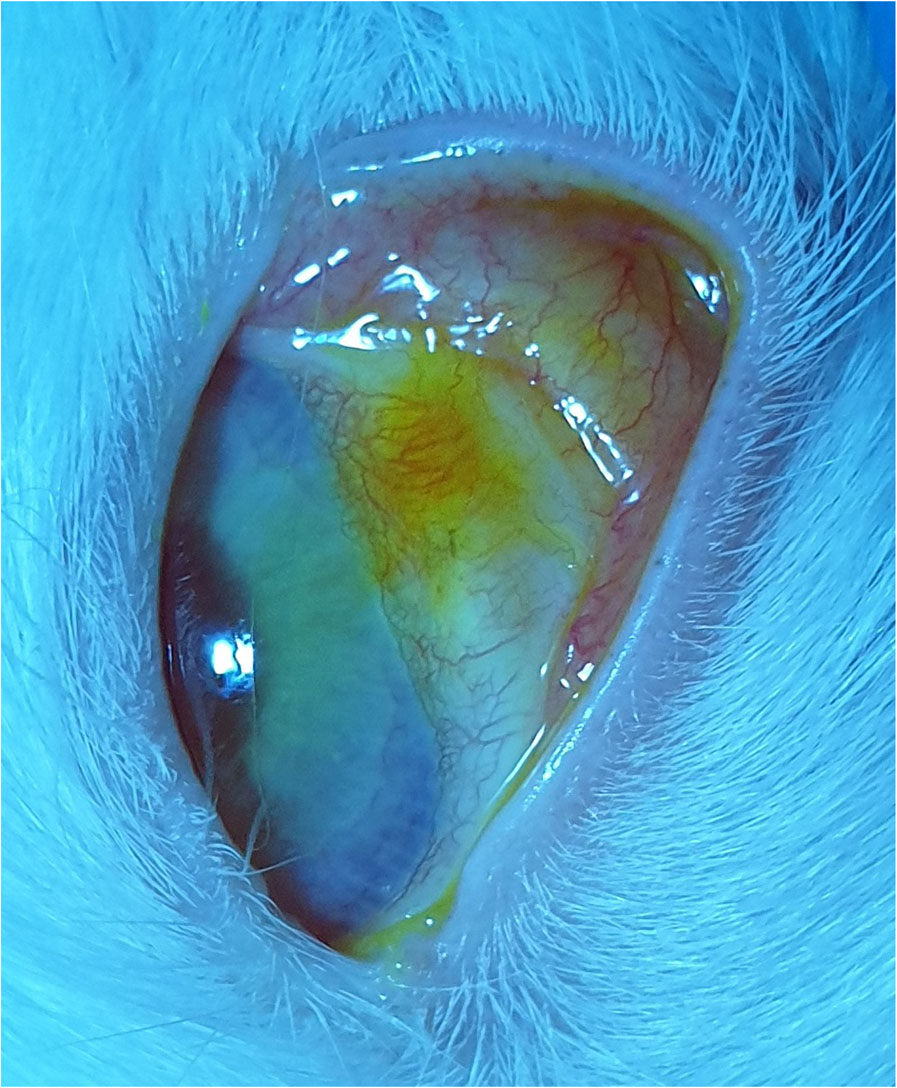
Evaluation of the ocular surface after 48 h of plasma exposure. External examination revealed conjunctival epithelial defect accompanied by mild redness and lid swelling during the first 48 h post-exposure

The external examination revealed some conjunctival epithelial defects at locations exposed to the plasma, mild redness, and lid swelling during the first 48 h. There were no findings of chemosis, severe redness, and discharge due to plasma exposure (Fig. 2).

The clinical observation after 6 months showed that the cornea remained clear in all groups.

**Fig. 3:**
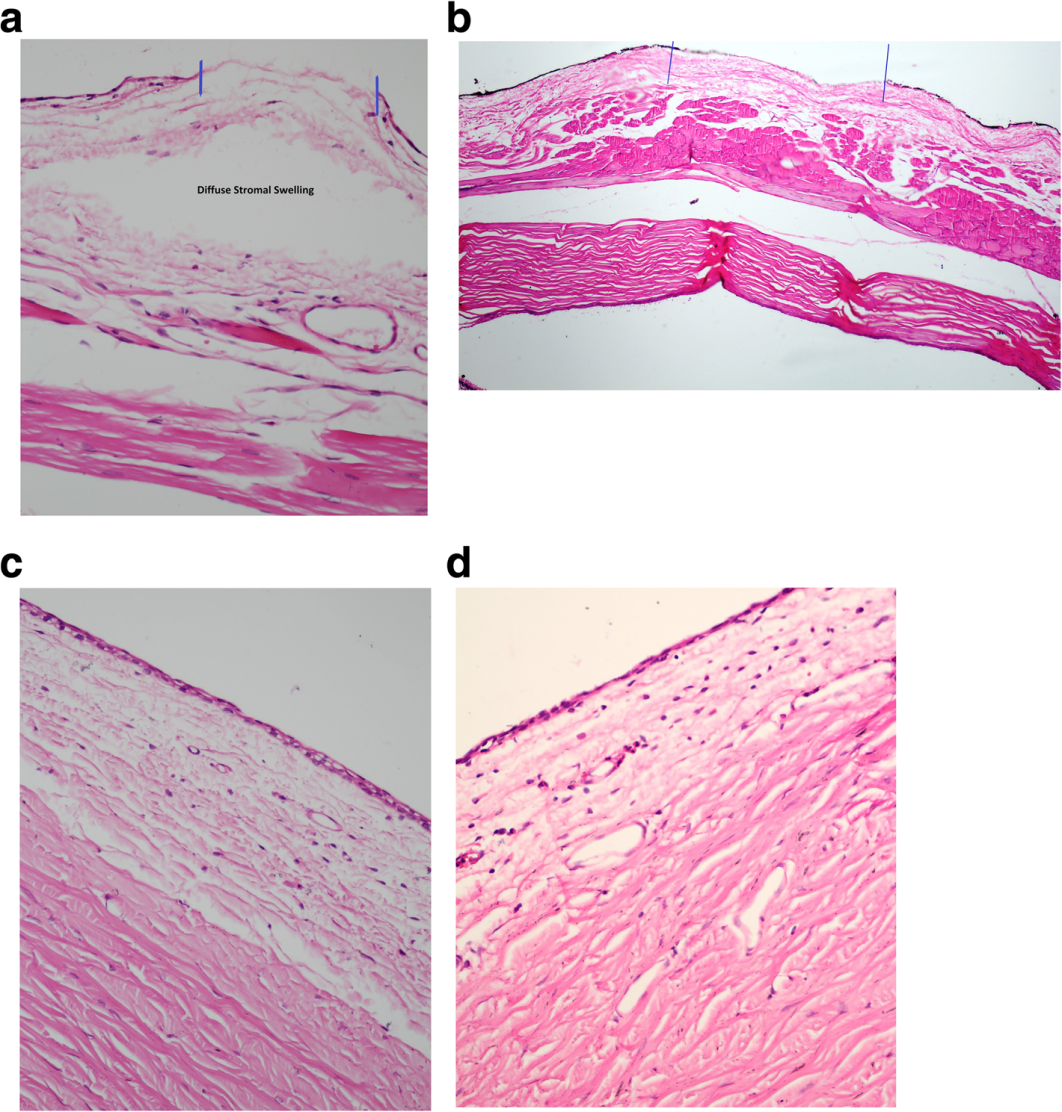
H&E-stained histological section of conjunctiva shows a slightly decreased vascularity, a diffused stromal swelling with extensive separation of collagen fibers, and a superficial epithelium loss following one week of the plasma exposure (a) [original magnification × 100 (H&E)]. The progression of re-epithelialization with subsidence of edema was noticed in one-month post-exposure (b) [original magnification × 100 (H&E)]. Histological sections after three months (c) [original magnification × 400 (H&E)] and six months (d) [original magnification × 400 (H&E)] post-exposure show that re-epithelialization is completed, stromal edema is resolved, and the conjunctival vascularization is restored. Of note, conjunctiva has returned to its normal histological order with no signs of chronic inflammation, hemorrhage, scar tissue, atypia, and dysplasia in the plasma exposed section. *Two blue lines show the plasma exposed areas of the conjunctiva

**Fig. 4:**
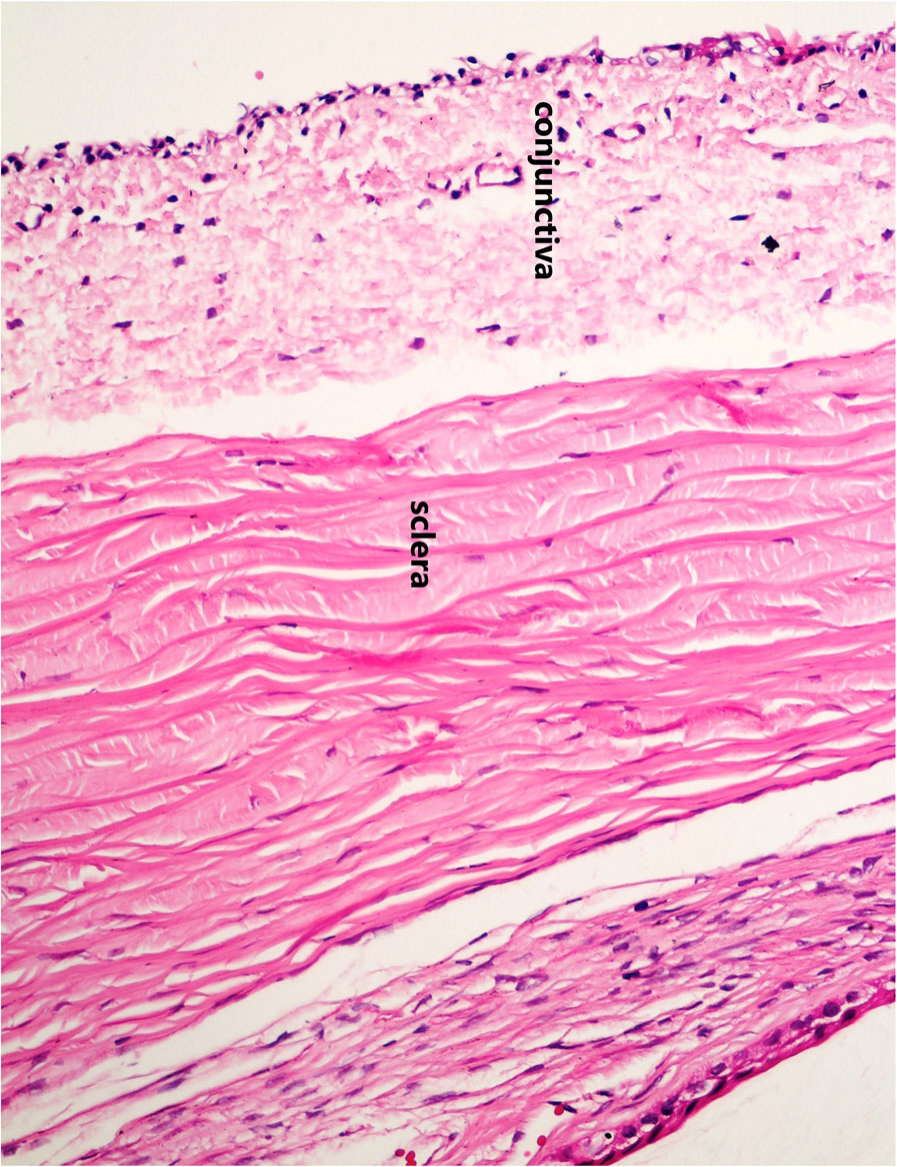
Normal histological order with no signs of any abnormalities is evident in the H&E- stained histological section of the conjunctiva in the control group (original magnification × 400 H&E)

Examination of H&E-stained histological sections of the conjunctiva in group A using a light microscopy revealed a slightly decreased vascularity, a diffuse stromal swelling with marked separation of collagen fibers, and some defects in the superficial epithelium (Fig. 3a). However, re-epithelialization progress was observed in addition to subsidence of edema in the histological section of the conjunctiva in group B with no scar tissue, deposition, or hemorrhage (Fig. 3b). In group C, the histological section of plasma-exposed conjunctiva revealed a complete re-epithelialization, total subsidence of stromal edema, and restored vascularization of the conjunctiva. Conjunctiva returned to its histological order with no sign of chronic inflammation, hemorrhage, scar tissue, atypia, and dysplasia (Fig. 3c). As illustrated in Fig. 3d, group D showed no abnormalities in the histological sections of conjunctiva following 6 months of plasma exposure (similar to the same area in the control eye (Fig. 4).

**Fig. 5:**
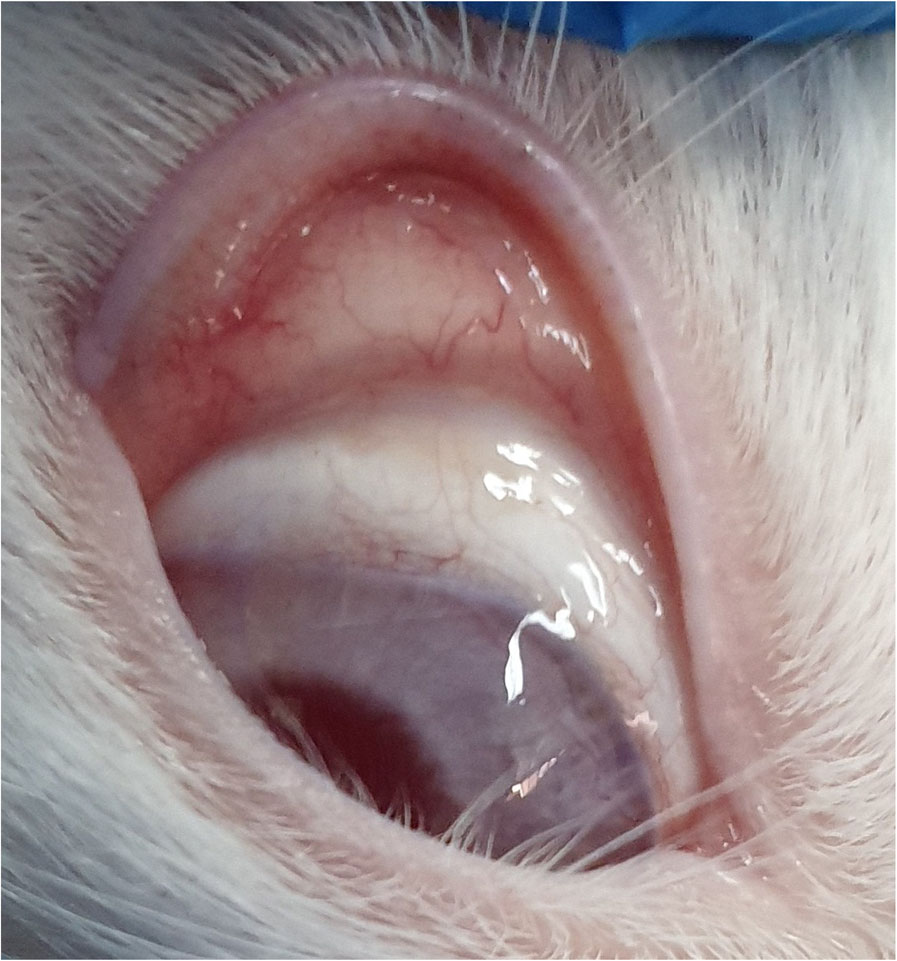
The plasma spots exposed areas on the rabbit’s eye were disappeared after 3 months of plasma exposure

As is displayed in Fig. 5, the plasma spot-exposed areas of the conjunctiva were not visible after 3 months.

## Discussion

The recent fast growth of plasma medicine and wide applications of ALTP in various medical fields, including wound healing, aesthetic surgery, ophthalmology, and even cancer therapy has led to vast studies in vitro, ex vivo, and in vivo (both in animal models and human subjects) [14]. In addition, safety has been always a major concern in medical researches. Then, before considering the use of ALTP in ophthalmology, the effects of plasma on the ocular tissues needed to be investigated. Brun et al. (2012) studied the safety and efficacy of cold atmospheric plasma (CAP) to inactivate ocular pathogens. According to their findings, the short-term application of CAP (for 0.5–5 min) could act as an effective and rapid ocular disinfectant for bacteria and fungi without significant damage to the ocular cells and tissues [15]. In another study, Alhabshan et al. (2013) examined the effect of CAP on wound healing in animal models after the corneal epithelial and basement membrane ablation [16]. They showed that CAP application to the cornea led neither to any evident adverse effects such as scar formation nor to any corneal wound healing. Reitberger et al. (2018) investigated the potential of argon cold plasma to treat corneal infections and its side effects on the viability of human corneal limbal epithelial cells [17]. Their results showed that cells treated for 0.5 to 5 min could completely recover after 24 h without changes in morphology. The efficacy of ALTP on the treatment of keratitis has been reported in several similar studies [18–21].

Also, a new approach to treating conjunctival cysts using ALTP has been introduced by Nejat et al. [22]. They used the Plexr device for removing cysts ranged from 2.1 to 4.8 mm. Their results showed no significant postoperative complications and cysts were completely healed without any recurrence during the follow-up. In another study conducted by the same team [23], plasma spots were used to treat the grade 3 and 4 CCh in six patients. Their results after 30 days, showed no ocular complications and in all treated eyes, grade 3 and 4 CCh were decreased to grade 1 and 2, respectively. They stated that these decreased grades of CCh were stable in the 6-month follow-up.

Another application of ALTP in ophthalmology is non-surgical blepharoplasty. The results of a study conducted by Tsioumas et al. (2017) suggest that the application of Plexr as a plasma generator device can be considered as a proper alternative soft surgery in blepharoplasty [7]. Rossi et al. (2018) evaluated the clinical results and collagen modification of the upper eyelid dermatochalasis after treatment, using Plexr [24]. They indicated that the plasma shows a collagen remodeling effect on the upper lid without any serious adverse effect. Plexr has been also applied in human aesthetic medicine for treating acne [25], treating the epidermoid cysts [26], reducing the wrinkles [10], rejuvenating [9], and other benign skin conditions [27]. The most advantages of this device include the little contraindications, minimal intraoperative pain, rapid treatment, and fast recovery with less cost which make it a safe and fast instrument for therapeutic purposes [28].

Based on the above-mentioned bits of knowledge, we hypothesized that the plasma technology can be used for the treatment of some ocular surface disorders. Then, as our first step, we conducted an animal study on the safety evaluation of plasma that showed no serious complications for plasma on the conjunctival tissue after one-month intervention [13]. In the present study, for achieving more reliable results, the histopathological changes of the conjunctiva were examined after 6 months of exposure to plasma. At the end of the examination, the histological findings under the light microscopy using H&E staining of conjunctiva showed no evidence of inflammation, edema, scar tissue, hemorrhage, atypia, and dysplasia. These observations are consistent with the absence of haze and inflammation in the clinical examination (see the additional file 1 and Fig. 5).

It should be noted that the present study faced the limitation of small number of experimental animals and limited follow-up. Therefore, further studies with higher number of animal models and a long-term follow-up period are suggested for more precise conclusions. Also, clinical trial studies are required for extending the application of Plexr for further human treatments. Another limitation of the present study was using the animal models instead of humans which can give rise to the misleading results.

## Conclusion

According to the histopathological findings, no adverse effects were observed on the conjunctival tissue after plasma exposure. Finally, it can be said that plasma soft surgery can be used as a minimally invasive, simple, and office-based technique to treat some ocular surface disorders.

## Supplementary Information

**Additional file 1.** A movie of applying plasma on four areas located at the superior part of rabbit conjunctiva and its effects six months later (File type: MP4).

## Data Availability

The datasets used and/or analyzed during the current study are available from the corresponding author on a reasonable request.

## Abbreviations

ALTP: Atmospheric-pressure low-temperature plasmas
H&E: Hematoxylin and eosin
CAP: Cold atmospheric plasma
CCh: Conjunctivochalasis
